# Effects of the High-Intensity Early Mobilization on Long-Term Functional Status of Patients with Mechanical Ventilation in the Intensive Care Unit

**DOI:** 10.1155/2024/4118896

**Published:** 2024-03-22

**Authors:** Chuanlin Zhang, Xueqin Wang, Jie Mi, Zeju Zhang, Xinyi Luo, Ruiying Gan, Shaoyu Mu

**Affiliations:** ^1^Department of Critical Care Medicine, The First Affiliated Hospital of Chongqing Medical University, Chongqing, China; ^2^School of Nursing, Chongqing Medical University, Chongqing, China; ^3^School of Nursing, Chongqing Medical and Pharmaceutical College, Chongqing, China

## Abstract

**Objective:**

Intensive care unit (ICU)-acquired weakness often occurs in patients with invasive mechanical ventilation (IMV). Early active mobility may reduce ICU-acquired weakness, improve functional status, and reduce disability. The aim of this study was to investigate whether high-intensity early mobility improves post-ICU discharge functional status of IMV patients.

**Methods:**

132 adult patients in the ICU who were undergoing IMV were randomly assigned into two groups with a ratio of 1 : 1, with one group received high-intensity early mobility (intervention group, IG), while the other group received conventional treatment (control group, CG). The functional status (Barthel Index (BI)), capacity of mobility (Perme score and ICU Mobility Scale (IMS)), muscle strength (Medical Research Council sum scores (MRC-SS)), mortality, complication, length of ICU stay, and duration of IMV were evaluated at ICU discharge or after 3-month of ICU discharge.

**Results:**

The patient's functional status was improved (BI scores 90.6 ± 18.0 in IG vs. 77.7 ± 27.9 in CG; *p*=0.005), and capacity of mobility was increased (Perme score 17.6 ± 7.1 in IG vs. 12.2 ± 8.5 in CG, *p* < 0.001; IMS 4.7 ± 2.6 in IG vs. 3.0 ± 2.6 in CG, *p* < 0.001). The IG had a higher muscle strength and lower incidence of ICU-acquired weakness (ICUAW) than that in the CG. The incidence of mortality and delirium was also lower than CG at ICU discharge. However, there were no differences in terms of length of ICU stay, duration of IMV, ventilator-associated pneumonia, and venous thrombosis.

**Conclusions:**

High-intensity early mobility improved the patient's functional status and increased capacity of mobility with IMV. The benefits to functional status remained after 3 month of ICU discharge. Other benefits included higher muscle strength, lower incidence of ICUAW, mortality, and delirium in IG.

## 1. Introduction

Patients with invasive mechanical ventilation (IMV) in the intensive care unit (ICU) are generally treated with sedation, so their activities are limited, and they often receive passive position changes by nurses and physiotherapists [1–4]. A cross-sectional survey involving 444 ICUs showed that only 57% of ICUs implemented early mobilization and 24.9% would evaluate and carry out it within 48 h after ICU admission [5]. Prolonged bed rest and immobilization may lead to complications such as muscular wasting, delirium, and ICU-acquired weakness (ICUAW) [6–8]. Previous studies had shown that more than 50% of patients with ICUAW were mechanically ventilated, and the incidence of ICUAW in patients with mechanical ventilation (MV) for 5 to 7 days was 25% to 65% [9], and that in patients with long-term MV (≥10 days), the incidence of ICUAW was over 67% [10]. In addition, ICUAW is associated with an increased risk of prolonged hospitalization, impaired recovery, and death [11]. Even among surviving discharged patients, the health-related quality of life is poor [12]. Several guidelines or expert consensus indicate that it is safe and feasible to initiate early activity in patients receiving IMV [1, 13–15]. Moreover, early mobilization should be implemented with daily goal planning by a multidisciplinary team [16]. However, translating research into clinical practice is challenging, especially for early activity in patients with IMV. There have been several studies of patients with IMV in the ICU, most of which report inactivity or more passive activity, and one study shows that half of the patients engaged in ambulation [17–20].

There have been conflicting results in studies examining the effects of early mobility on functional status and quality of life in the ICU [21–29]. Studies have focused more on the effect of early activity on reducing ICUAW than on short- and long-term functional status after ICU discharge [30–34]. If the patients have good physical function after ICU discharge, it may reduce the care burden of the family members. Therefore, it is necessary to understand the impact of early activities on the physical function postdischarge of patients. An international prospective cohort study reports that high-intensity early mobilization is an independent predictor of patient's independent living ability after ICU discharge [35]. Other studies have also suggested that the intensity of early mobilization may influence outcomes, but further high-quality research is needed [36, 37]. Moreover, few randomized controlled studies were conducted to detect the effects of high-intensity early mobilization to improve the patient's physical functional and independent living ability in IMV patients in the ICU.

The aim of this study was to investigate whether high-intensity early mobility improves the patient's functional status compared to conventional treatment at 3 months of ICU discharge. The secondary objectives were to investigate the effects of high-intensity early mobilization on the capacity of mobility, muscle strength, ICUAW, delirium, in-ICU mortality, 3-month mortality, ventilator-associated pneumonia (VAP), venous thrombosis (VTE), length of ICU stay, and duration of MV.

## 2. Methods

### 2.1. Study Design

This study was a parallel randomized controlled trial to investigate the effects of the high-intensity early mobility on the patient's functional status. Informed written consent was obtained from all participants or their responsible family member. Ethical approval was obtained from the Ethics Committee of the First Affiliated Hospital of Chongqing Medical University (Document No. 2018-015). A CONSORT checklist was used to guide the reporting of the research.

### 2.2. Setting and Participants

Patients eligible for recruitment were aged 18 years or older that were expected to be on IMV for >24 hours at the time of screening. The Barthel score at two weeks before ICU admission was estimated from the patient's family members, and patients were included if they had an independent functional status with a Barthel index (BI) [38] of 100 points. Patients were excluded if (1) cognitive impaired with an inability to understand command and mobility; (2) contraindications for mobilization [1]; (3) death within 48 hours of admission to ICU; (4) ICU admission for the second time in the last month; (5) MV time >48 hours in the outside hospital before ICU admission; and (6) lower limb mobility disorder.

### 2.3. Study Procedure

In accordance with the principle of 1 : 1, the computer randomly generated 132 random numbers, which were written on paper blocks and placed in an envelope. Patients who met the inclusion criteria were randomly assigned to the IG or CG according to the numbers selected from a paper block by the investigator. In this study, the group assignment, patients, and outcomes assessors were blinded. A data collection form was developed, and only the random number was displayed. The outcomes assessors were unaware that the patients were in the experimental or control group and recorded the results in the specific worksheet. The assessor of the main outcomes was the same individual. However, due to the characteristics of the intervention measures, intervention implementers could not be blinded. The research team included ICU trained doctors, nurses, and physiotherapists. The CG received conventional treatment performed by physiotherapists, including position changes, passive range of motion, cycle, and physiotherapy (bed mobility, transfer training, and balance training). The start time and level of mobility for each patient were decided by physiotherapists. The IG received the same level of clinical care as CG except for the high-intensity early mobility program after enrolment within up to 48 hours of ICU admission. Schujmann et al.'s research content was taken into consideration when we made the high-intensity early mobility program [21]. Each morning, the research team assessed the patients and set a target activity level for the day. The intensive activity level included exercise and posture changes: level 1 (passive activity), level 2 (sitting in bed, responsive to instruction and muscle strength ≤2), level 3 (sitting in chair, responsive to instruction and muscle strength ≥3), level 4 (standing), and level 5 (ambulation). The details of the activities are shown in Table 1. Both groups underwent conventional physical therapy in the morning. The CG patients also received an afternoon conventional therapy session, and IG patients underwent high-intensity early mobility in the afternoon, five times a week (Monday through Friday). The variance between groups was just the intensity of the early mobility program (i.e., the IG did high-intensity early mobility, while the CG did early mobility at an intensity as per the treating therapist discretion). For patients with sedative agents, the goal is adjusting to reach the Richmond Agitation Sedation Scale score of −1 to +1. The maximum activity level would be recorded by researchers every day.

The criteria for initiation and continuation of the high-intensity early mobility program follow the safety index described in the expert consensus [1], and some points were as follows: respiratory rate (RR) ≤30 breaths per minute, no arrhythmias or acute ischemia, heart rate (HR) between 60 and 120 beats per minute, no use or increased dose of vascular drugs, mean arterial pressure (MAP) between 65 and 120 mmHg, no active bleeding, and no prescription of bed rest. When patients were treated with IMV, respiratory oxygen inspiratory fraction (FiO_2_) ≤60% and positive end expiratory pressure (PEEP) ≤10 cmH_2_O [39]. Adverse events were recorded during early mobility including falls, unplanned extubation or any invasive tube shedding, and cardiac arrest.

### 2.4. Outcomes

The primary outcome was the patient's functional status evaluated by BI after ICU discharge. The BI score was followed up by telephone 3 months after ICU discharge. Patients were divided into two groups by functional status based on whether BI score ≥85 [40, 41]. The secondary outcome included capacity of mobility and muscle strength. The capacity of mobility was assessed by the Perme score [42], and ICU Mobility Scale (IMS) [43]. The score of the Perme score and IMS were collected and documented at ICU discharge. Muscle strength was evaluated by the Medical Research Council sum scores (MRC-SS) at enrolment and discharge from ICU, and MRC-SS ≤48 was defined ICUAW [44, 45]. Other secondary outcomes including the patient's ICU length of stay, in-ICU mortality, 3-month mortality, the incidence of complication (VAP, VTE, and delirium), and duration of IMV were also recorded. Delirium incidence was evaluated three times a day (morning, midday, and evening) with the Confusion Assessment Method for the ICU (CAM-ICU) [46]. Although our data collection window was during the global coronavirus pandemic, our hospital was not a designated hospital for treating COVID-19 patients, so the ICU services were minimally impacted by the workload, and not altered work practices associated with the pandemic response. There were few potential limitations or implications for the study findings.

### 2.5. Sample Size

The sample size calculation was based on Schujmann et al. [21]. The difference in the means of the primary outcome (BI score) was expected to be 13 points, with a standard deviation of 22 points. The G^*∗*^Power software [47] (Version 3.1.9.4) (RRID: SCR_013726; https://www.gpower.hhu.de/) was used to calculate the number of subjects as 48 per group for a total of 96 patients in this study, using a statistical power of 80% and an *α* error of 0.05.

### 2.6. Statistical Analysis

All data were analyzed using SPSS (version 21; IBM Corp, Armonk, New York, USA). General data were expressed as the mean ± standard deviation, percentage, or median (quartile). Intention-to-treat analysis was performed for all enrolled patients. General data comparisons between the two groups were performed using Student's *t*-test, a Mann–Whitney nonparametric test, and a chi-square test as appropriate. The comparisons of intensity of physiotherapy, functional status, MRC-SS, length of ICU stay, and duration of MV between groups were conducted by Student's *t*-test or Mann–Whitney nonparametric test. Difference of mortality, delirium, VAP, VTE, and ICUAW rate were compared using a chi-square test.

## 3. Results

### 3.1. Demographics

**Figure 1:**
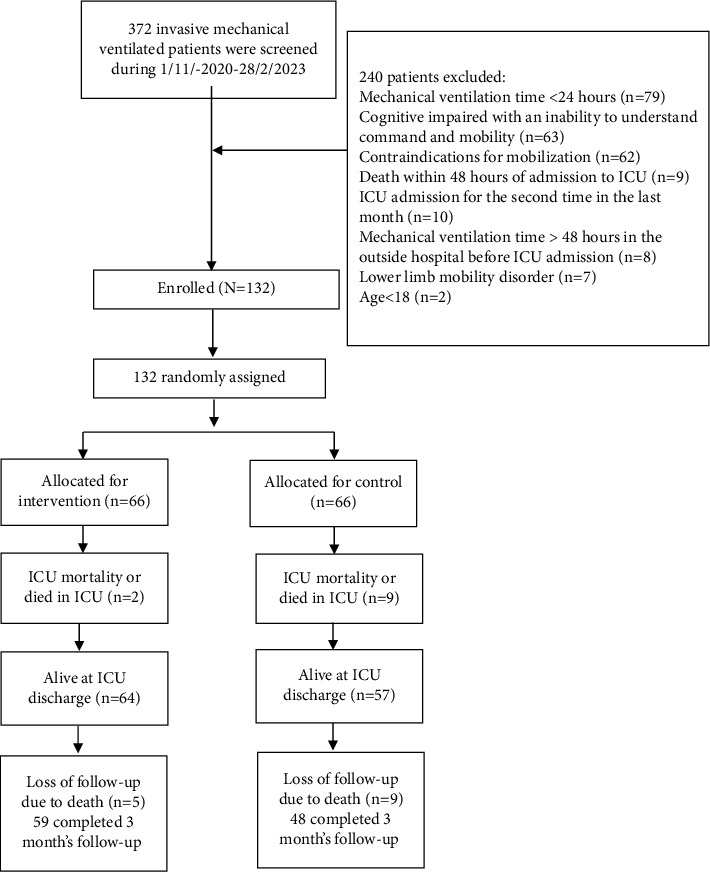
Consort flow diagram of this study procedure.

As shown in Figure 1, 372 patients with IMV were screened from November 1, 2020, to February 28, 2023, and 132 patients were enrolled and randomly assigned to two groups (IG = 66 and CG = 66). The enrolled 132 patients (84 males and 48 females) aged 18 to 95 years older (60.4 ± 17.8 years). The mean body mass index was 22.1 ± 2.7, and APACHE II was 22.1 ± 6.8. There was no statistical difference in baseline data between the two groups except for type 2 diabetes (Table 2).

### 3.2. Primary Outcome Analysis

There were two deaths in the IG and nine deaths in the CG at ICU discharge. As a result, only 121 patients were included in the discharge phase data analysis. The BI scores in the IG were higher than those in CG at 3-month post-ICU discharge (90.6 ± 18.0 vs. 77.7 ± 27.9; *p*=0.005). According to BI scores, patients were divided into two groups, and it was found that the number of patients with functional independence (with BI scores ≥85) in the IG was higher than that in the CG (76.3% vs. 56.3%; *p*=0.028) (Table 3).

### 3.3. Secondary Outcome Comparison between Two Groups

The intensity of physiotherapy in the CG group accounted for 50% at level 1∼level 2, while the percentage in the IG group was superior at level 4∼level 5 (62.1% vs. 24.2%). There were significant differences between the mean intensity of physiotherapy received between the two groups (3.7 ± 1.1 vs. 2.6 ± 1.2; *p* < 0.001). Patients in the IG had a higher Perme score (17.6 ± 7.1 vs. 12.2 ± 8.5; *p* < 0.001), IMS (4.7 ± 2.6 vs. 3.0 ± 2.6; *p* < 0.001), and MRC-SS (50.3 ± 11.7 vs. 37.3 ± 18.6; *p* < 0.001), respectively. The incidence of ICUAW was lower in IG than that in CG (17.2% vs. 54.4%; *p* < 0.001). The incidence of delirium and in-ICU mortality was lower in IG than that in CG (18.2% vs. 36.4% *p*=0.019; 3.0% vs. 13.6% *p*=0.027) (Table 4). There were no significant differences between the two groups in terms of duration of mechanical ventilation, length of ICU stay, VTE, VAP, and in 3-month mortality (Tables 3 and 4). No adverse events which required additional treatment were reported between the two groups.

## 4. Discussion

This randomized controlled trial investigated the effects of high-intensity early mobility on patients with IMV in the ICU. The current study results showed that compared with the CG, high-intensity early mobility improved functional status and the functional independence after 3 months of ICU discharge, the capacity of mobility, and increased muscle strength at ICU discharge. The incidence of ICUAW, delirium, and in-ICU mortality was lower than that in the CG.

Compared to previous studies, the novelty in this present study was that our patients enrolled were sicker patients (higher APACHE II score 22.1 ± 6.8) and all were on IMV. High-intensity early mobility is a known difficult procedure to perform in the ICU, thus necessitating this study to be conducted to ascertain the benefit of a difficult intervention [22–24, 48]. Moreover, early implementation of mobilization was one of the key factors for success, in addition to goal-directed and gradual administration of highly intensive early mobility. In this study, patients were screened and enrolled 24 hours after ICU admission, and the high-intensity early mobilization was conducted based on the status of patients. Similarly, Liu et al. [49] and Ding et al. [50] found that patients with sepsis may benefit most from early activity starting from 2 to 4 days. A systematic review and meta-analysis showed that systematic early mobilization within 7 days of admission to ICU improved physical function, functional status, and walking ability compared with late mobilization [51].

In this study, the primary outcome BI scores were higher in the IG than those in the CG, and the proportion of patients with functional independence was also higher. The findings from this present study prove that, despite high-intensity early mobility being a difficult procedure to perform, it will be a worthwhile clinical intervention to significantly improve functional independence without significant risk of harm to the patients. Indeed, the present study data showed that half of the patients in the CG performed intensity of physiotherapy at level 1∼level 2, and only 24.2% performed out of bed activity (level 4∼level 5), compared to 62.1% in the IG. Similarly, in a multicentre, parallel-group randomized controlled trial including 308 patients, more than 98% were on IMV and those who received intensive physical rehabilitation therapy had an improved functional independence after 3 months [22]. In addition, Watanabe and colleagues [37] found high-dose early rehabilitation can improve the daily activity ability of patients with mechanical ventilation in ICU.

This study also found that the MRC-SS in both groups was improved compared with those at ICU admission, and the improvement was more obvious in the IG at ICU discharge. A higher level of mobility in IG may change the course of function loss. As a result, ICUAW was only 17.2% in the IG and 54.4% in the CG. The present study findings corroborated with a previous systematic review and meta-analysis [52]. ICU-acquired weakness is a common complication of critical illness, with an estimated incidence of 25∼50% [53, 54]. Sidiras et al. [55] found that the quality of life and functional independence of patients with ICUAW at 3 and 6-month post-ICU discharge were worse than those without ICUAW, but at 6 months, there was no significant difference in muscle strength between the two groups. This present study followed up 42 patients with ICUAW at ICU discharge for 3 months and found that both the IG and CG had a lower BI score. Therefore, attention should be paid to the patients with ICUAW at ICU discharge (Table 5).

For short and long-term mortality, the results varied from studies [23, 35, 52, 56]. The present study results were similar to Zayed et al. [56] and Scheffenbichler et al. [35] that the high-intensity early activity reduced short-term mortality, but there was no difference in 3-month mortality or longer after discharge. Hodgson and colleagues [26] also found that an increase in early active mobilization in patients with IMV in the ICU did not change the median alive time at 180 days. Delirium is a common complication in the ICU, especially in patients with MV, and inactivity is a known risk factor [57]. Two systematic review and meta-analysis studies have shown that early mobilization or physical activity interventions can reduce the incidence or duration of delirium [58, 59]. Similar to outcomes in other studies [23, 29, 60], the incidence of delirium was lower in IG patients who received high-intensity early mobilization than in CG patients who received conventional treatment. In addition, implementation of early mobilization can reduce the incidence of delirium, which may be related to the intervention time. Nydahl et al. [46] found that activity in the evening could not reduce the incidence of delirium for ICU patients.

The findings of this present study have several implications for clinical practice. First, the high-intensity early mobility is safe and feasible for patients with IMV and can improve the patient's functional status, capacity of mobility, and muscle strength. Second, multidisciplinary teamwork at least including ICU physicians, nurses, and physiotherapists is needed in high-intensity early mobility. Third, this present study suggests early mobilization should be evaluated and performed as early as 24 hours after ICU admission for those without contraindication, including different intensive activities, especially early active mobility and out-of-bed activity. Finally, the findings of the present study also suggest whether in ICU or post-ICU discharge active and passive activities should be carried out early for patients with ICUAW.

This study has several limitations. First, the functional recovery measurement of alive at ICU discharge was limited to BI scores, and the indicators of return to work, cognitive function, and health-related quality of life were not included. The main consideration is that patients with IMV have more serious diseases, fewer patients may return to work 3 months after ICU discharge, and the average age of this study population was 60.4 years, so the indicators of return to work are of little significance. More insights are wanted to be known about patients' daily living ability and whether they could live independently, so as to reduce the burden of family care. Second, patients in the CG have a higher mortality rate before ICU discharge, which may lead to information bias in the analysis of patients' functional status. However, when both groups of patients who died before ICU discharge were included in intention-to-treat analysis, similar results for the primary outcome were still found. Third, only the maximum intensity of early mobility achieved by patients was recorded but not the duration of activity. Because the main purpose is to understand whether high-intensity early mobility improves post-ICU discharge functional status of IMV patients. The effect of early mobility dose (both intensity and duration) on the primary outcome of IMV patients was not investigated in the present study.

## 5. Conclusions

In summary, the high-intensity early mobility in patients with IMV is safe and feasible, which improves the patient's functional status and the number of patients with functional independence at 3-month post-ICU discharge. Meanwhile, it improves the capacity of mobility and muscle strength and decreases the incidence of ICUAW, delirium, and mortality during ICU stay. For patients with ICUAW, active and passive activities should be carried out as early as possible, mainly active mobility.

## Figures and Tables

**Table 1:** High-intensity early mobility program for patients with invasive mechanical ventilation.

Level	Defined	Exercise and postures changes
Level 1	Passive activity	① 20 min: passive cycle ergometer for lower limbs
		② Passive range of motion in lower and upper limbs
		③ Passive position change

Level 2	Sitting in bed (responsive to instruction and muscle strength ≤ 2)	① 15 min: assisted cycle ergometer for lower limbs
		② Assisted exercises for upper limbs
		③ Assisted position changes in bed
		④ 30 min: assisted in bed sitting

Level 3	Sitting in chair (responsive to instruction and muscle strength ≥ 3)	① 15 min: assisted cycle ergometer for lower limbs
		② Resisted upper and lower limb exercises
		③ 30 min: assisted or active bedside sitting
		④ Sitting in chair

Level 4	Standing	① 10 min: assisted cycle ergometer for lower limbs
		② Resisted upper and lower limb exercises
		③ Active bedside sitting
		④ Standing with assistance

Level 5	Ambulation	① Resisted upper and lower limb exercises
		② Active bedside sitting
		③ Standing
		④ Ambulation with assistance (worker or device)

**Table 2:** General information of the enrolled patients.

Variable	Intervention group (*n* = 66)	Control group (*n* = 66)	*t/x* ^2^	*p*
Age (y)	60.5 ± 16.9	60.3 ± 18.7	0.044	0.965
Male	37 (56.1)	47 (71.2)	3.274	0.070
Body mass index	22.5 ± 3.0	21.7 ± 2.4	1.662	0.099
Diagnosis category			1.423	0.759
Medical	48 (72.7)	52 (78.8)		
Surgical	12 (18.2)	11 (16.7)		
Obstetric and gynaecologic	3 (4.5)	1 (1.5)		
Others	3 (4.5)	2 (3.0)		
APACHE II score	22.4 ± 6.8	21.7 ± 6.8	0.603	0.548
Sepsis	20 (30.3)	13 (19.7)	1.980	0.159
Coronary heart disease	10 (15.2)	7 (10.6)	0.608	0.436
Hypertension	18 (27.3)	18 (27.3)	0.000	1.000
Type 2 diabetes	24 (36.4)	12 (18.2)	5.500	0.019
Liver disease	7 (10.6)	4 (6.1)	0.893	0.345
PAOD	6 (9.1)	7 (10.6)	0.085	0.770
Artificial airway type			0.075	0.784
Tracheal intubation	59 (89.4)	58 (87.9)		
Tracheotomy tube	7 (10.6)	8 (12.1)		
Vasoactive drugs	45 (68.2)	38 (57.6)	1.590	0.207
MRC-SS	34.3 ± 11.4	31.5 ± 10.4	1.453	0.149
Capacity of mobility				
ICU mobility scale	1.1 ± 1.0	1.0 ± 1.0	0.171	0.864
Perme score	7.0 ± 4.1	6.2 ± 3.7	1.263	0.209

APACHE II, acute physiology and chronic health evaluation II; MRC-SS, Medical Research Council sum score; PAOD, peripheral arterial occlusive disease. Data described in mean ± SD, absolute number (%).

**Table 3:** Follow-up data analysis of patients at 3-month post-ICU discharge.

Variable	Intervention group (*n* = 59)	Control group (*n* = 48)	*t*/*x*^2^	*p*
Barthel index	90.6 ± 18.0	77.7 ± 27.9	2.762	0.005
% of independent patients	76.3	56.3	4.820	0.028
3-month mortality	5 (7.8)	9 (15.8)	1.875	0.171

Data described in mean ± SD, absolute number (%).

**Table 4:** Comparison for outcomes between two groups.

Variable	Intervention group (*n* = 66)	Control group (*n* = 66)	*t*/*z*/*x*^2^	*p*
Intensity of physiotherapy			23.392	<0.001
Level 1∼level 2	10 (15.2)	33 (50.0)		
Level 3	15 (22.7)	17 (25.8)		
Level 4∼level 5	41 (62.1)	16 (24.2)		
Mean intensity of physiotherapy	3.7 ± 1.1	2.6 ± 1.2	5.353	<0.001
ICU mobility scale	4.7 ± 2.6	3.0 ± 2.6	3.836	<0.001
Perme score	17.6 ± 7.1	12.2 ± 8.5	3.980	<0.001
MRC-SS	50.3 ± 11.7	37.3 ± 18.6	4.785	<0.001
ICU-acquired weakness	11 (17.2)	31 (54.4)	18.408	<0.001
Length of ICU stay (d)	12.5 (5.8, 21.0)	12 (8.0, 26.0)	−0.859	0.390
Duration of mechanical ventilation (d)	6.7 ± 4.7	8.9 ± 8.0	−1.935	0.055
Delirium incidence	12 (18.2)	24 (36.4)	5.500	0.019
Venous thrombosis	11 (16.7)	12 (18.2)	0.053	0.819
Ventilator-associated pneumonia	3 (4.5)	4 (6.1)	0.000	1.000
In-ICU mortality	2 (3.0)	9 (13.6)	4.860	0.027

MRC-SS, Medical Research Council sum score; ICU-acquired weakness, defined by MRC-SS <48. Data described in mean ± SD, absolute number (%).

**Table 5:** Comparison of two groups of discharged patients with ICU-acquired weakness.

Variable	Intervention group (*n* = 11)	Control group (*n* = 31)	*t*/*x*^2^	*p*
3-month mortality	1 (9.1)	7 (22.6)	0.283	0.595
Barthel index	65.5 ± 29.0	64.5 ± 29.5	0.083	0.936
% of independent patients	20.0	29.1	0.016	0.900

Data described in mean ± SD, absolute number (%).

## Data Availability

The data that support the findings of this study are available from the corresponding author upon reasonable request.
